# Application of Chorionic Villus Sampling to Longitudinal Studies in Pregnant Non-Human Primate Models

**DOI:** 10.3390/ani16030374

**Published:** 2026-01-24

**Authors:** Sarah N. Cilvik, Michelle N. Sullivan, Theodore R. Hobbs, Jenna N. Castro, Brady M. Wessel, Henry F. Harrison, Victoria H. J. Roberts

**Affiliations:** 1 Department of Pediatrics, Wake Forest University School of Medicine, Winston-Salem, NC 27101, USA; sarah.cilvik@wfusm.edu; 2 Division of Comparative Medicine, Oregon National Primate Research Center (ONPRC), Oregon Health & Science University (OHSU), Beaverton, OR 97006, USA; sullishe@ohsu.edu (M.N.S.); hobbst@ohsu.edu (T.R.H.); 3 Division of Reproductive and Developmental Science, Oregon National Primate Research Center (ONPRC), Oregon Health & Science University (OHSU), Beaverton, OR 97006, USA; castroje@ohsu.edu (J.N.C.); wesselb@ohsu.edu (B.M.W.); harrihen@ohsu.edu (H.F.H.)

**Keywords:** rhesus macaque, placental biopsy, new approach methods, animal use reduction

## Abstract

The rhesus macaque (*Macaca mulatta*) is a valuable model for pregnancy research due to its physiological similarity to humans and the ability to conduct studies in a controlled environment. Our previous work used non-invasive imaging methods to assess placental hemodynamics across gestation with correlative tissue analysis post-delivery. Here, we expand access to longitudinal timepoints from ongoing pregnancies by obtaining placental biopsies using ultrasound-guided needle aspiration. This approach aligns with New Approach Methods (NAMs) and supports animal welfare by reducing the number of animals required. We describe a chorionic villus sampling (CVS) simulation model which facilitates training to gain proficiency in technical skills prior to performing the procedure on animals. We report outcomes from three rhesus macaques that underwent CVS three times between gestational days 40 to 106 (term: 165 days). Although biopsy samples are smaller than whole placenta, tissue yields were sufficient for multiple uses. We demonstrate (1) appropriate histology from aspirated samples, (2) good RNA quality and yield, and (3) the ability to isolate trophoblast organoids, an advancement in NAMs that reduces the need for first-trimester surgical delivery. No spontaneous preterm delivery occurred following serial CVS procedures, supporting the use of this sampling method to maximize animal utilization in longitudinal pregnancy studies.

## Simple Summary

This study addresses the limited ability to examine placental health during an ongoing pregnancy, restricting the understanding of how complications may arise from abnormal placental development or function over time. The objective was to trial a minimally invasive method for obtaining serial placental samples from pregnant rhesus monkeys using ultrasound guidance and a fine needle biopsy technique. A realistic training simulation model was developed for personnel to practice the technique before performing sample collection from live animals. The results showed that the tissue samples obtained by needle biopsy were of sufficient quality for detailed examination, including evaluation of tissue structure and genetic material, as well as placental cell culture in a laboratory setting. Importantly, repeated sampling did not cause injury or early pregnancy loss. These findings demonstrate a safe approach for pregnancy research in a clinically relevant animal model that improves scientific knowledge and maximizes animal involvement through serially sampling across the course of pregnancy, while supporting improved outcomes for maternal and fetal health.

## 1. Introduction

During pregnancy, inadequate placental development and subsequent dysfunction result in a range of adverse fetal and maternal outcomes [1,2,3,4]. The first trimester is a crucial time for the establishment of appropriate placentation, yet our current knowledge of early gestation is inadequate, and understanding of placental development and function throughout pregnancy is impeded in part by the lack of longitudinal access to the tissue. Non-human primates (NHPs) are excellent animal models for pregnancy studies as they have long gestational periods and a hemochorial placenta structure similar to humans [5]. NHP models enable highly translational longitudinal placental studies that improve our understanding of pregnancy and increase our ability to identify human pregnancies at risk for poor outcomes. We routinely perform non-invasive in vivo imaging using contrast-enhanced ultrasound and magnetic resonance imaging (MRI) to examine placental hemodynamics and the contribution of blood flow to placental function in NHP models [6,7,8,9,10,11]. Traditionally, stages of placental development have been studied by collecting placental tissues after fetectomy at specific developmental ages to perform correlative in vitro studies for the assessment of functional read-outs in these NHP models. This required surgical pregnancy termination in multiple animals to characterize longitudinal changes throughout gestation, and it precluded prospective studies to match offspring outcomes with pregnancy pathology. Furthermore, the research-driven need for early gestation NHP placenta samples has increased the request for pregnancy termination in the first trimester. An alternative strategy would be to perform a minimally invasive ultrasound-guided procedure to obtain placental biopsies by chorionic villus sampling (CVS). This approach would decrease the number of animal subjects required and reduce the inherent variability between subjects.

The CVS technique is a common procedure performed in pregnant women, but the application of this method to NHP studies to date has been limited. In clinical obstetric practice, CVS is used to acquire placental tissue for prenatal diagnostic testing [12,13,14]. The use of ultrasound guidance to track anatomical structures and landmarks has improved safety while performing minimally invasive procedures [15]. However, there is a need to adequately train operators as they learn new techniques. The pregnant uterus increases complexity in training endeavors with additional skill required to avoid causing harm to the fetus when a needle is introduced into the in utero environment. The complication rate of CVS varies within the available literature, but the reported rate of miscarriage is approximately 0.5% [16] with a transcervical approach having a slightly higher risk than transabdominal in human patient subjects [17]. In rhesus macaques, the presence of multiple colliculi in the cervical anatomy [18] creates a tortuous path that impedes biopsy via a transcervical approach, thereby making transabdominal biopsy the best option in this animal model. In humans, for prenatal diagnostic testing, very modest tissue amounts are needed (typically 5 mg [14,19]) and a cautious approach to minimize disruption of the placenta in situ is favorable to reduce the risk of adverse outcomes. For research purposes, the ability to collect a larger biopsy has the potential to facilitate multiple uses of the tissue in experimental analysis, yet care must still be taken not to perturb the placenta and jeopardize the pregnancy.

## 2. Materials and Methods

### 2.1. Simulation Model

**Figure 1:**
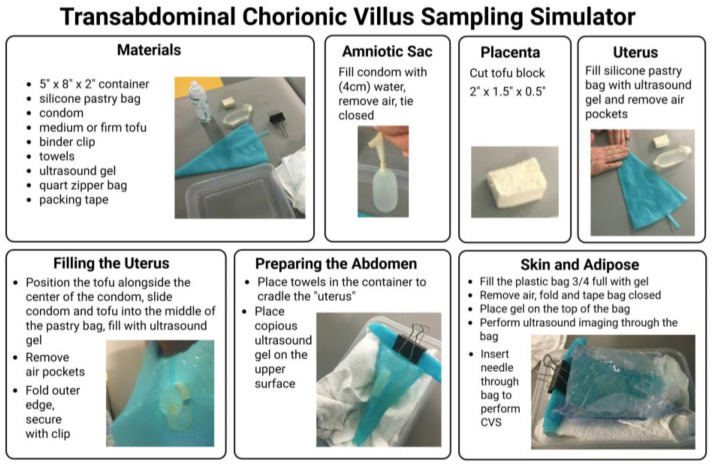
Transabdominal chorionic villus sampling simulation model. Step by step guide to the set-up of simulation apparatus to mimic the pregnant uterus for use as a CVS training aid [15].

The use of a simulation training model for CVS assumes some prior experience by veterinarians with ultrasound. This may be in the context of the identification and basic assessment of pregnancy or somewhat more advanced with performing minimally invasive procedures. The ultrasound-guided introduction of a needle into a body cavity and the tracking and placement of the needle tip requires manual dexterity and precise coordinated movements. Simulation training is effective for developing these skills. Figure 1 provides step by step instructions for the assembly of a CVS simulation model using materials to mimic the consistency of the maternal abdomen (zipper bag), uterus (silicone pastry bag), placenta (tofu block), and amniotic cavity (water-filled condom), as developed and described by Nitsche et al. [15]. For this study, the surgical veterinarians at the ONPRC (MNS and TH) utilized this simulator with training under the guidance of a board-certified neonatologist with extensive experience in performing CVS in NHPs (SNC) prior to performing the procedure in live animals. A GE Voluson 730 ultrasound unit with a 5–9 MHz sector probe was used for all simulated and live sampling procedures. The ultrasound probe was placed on the exterior of the apparatus and learners obtained a long segment view of the tofu “placenta” and underlying fluid filled “amniotic cavity”. After visualizing an optimal sampling location, they introduced an 18 or 20 gauge 3.5 cm spinal needle into the “placenta” in the plane of the ultrasound probe, maintaining visualization of the needle tip throughout insertion. Once the needle was observed within the block of tofu, the stylet was removed and a 5 mL syringe containing 1 mL of media was attached to the needle. Learners then performed gentle movement of the syringe in and out 5 to 10 times while applying 3–4 mLs of negative pressure to the syringe. The syringe and needle unit was removed from the “placenta” and expelled the extracted tofu “villi” into a dish. This task trainer allowed for skill acquisition and optimization at numerous steps within this challenging procedure, including visualization and manipulation of a needle under ultrasound guidance, as well as determination of how aspiration pressure and needle passage impact sample yield [20,21].

As a technical note, the use of a specifically designed aspiration syringe handle (Anthony Products Inc., Indianapolis, IN, USA Item No. WJ-20-0020 20cc) may increase operator comfort and generate more powerful suction to aid in dislodging fragments of placental tissue as they are drawn through the needle and into the media-filled syringe.

### 2.2. Animals

All experimental protocols were approved by the Institutional Animal Care and Use Committee (IACUC) of the ONPRC, and all procedures contributing to this work complied with the ethical standards of the Guide for the Care and Use of Laboratory Animals and the Animal Welfare Act and Regulations enforced by the United States Department of Agriculture. Female rhesus macaques (*n* = 3) used in this study were all healthy, normal weight (Table 1), and fed a standard chow diet (Purina, St. Paul, MN, USA, Lab Diet 5000. Animals were assigned to the Time-Mated Breeding program at the ONPRC and had a proven reproductive history with prior live birth offspring. All early pregnancies were identified through daily monitoring of estrogen and progesterone levels and confirmed by ultrasound. Based on extensive hormonal assessment studies conducted at the ONPRC [22,23], gestational age is calculated by considering the first day of conception as two days after the estrogen peak for consistent and reproducible dating.

### 2.3. Chorionic Villus Sampling

On the day of the procedure, following an overnight fast, animals were sedated with ketamine (10 mg/kg IM), followed by endotracheal intubation and anesthesia maintenance on 1–2% inhaled isoflurane gas mixed with 100% oxygen. Hair was clipped from the abdomen, and the area was sterilely prepped and draped for CVS. A transabdominal ultrasound-guided approach was used for direct insertion of an 18- or 20-gauge 3.5 cm spinal needle into the anterior placental disk. The longest axis of the placenta was targeted for needle advancement. Once the needle was inserted through the uterine wall and the needle tip was positioned within the placenta, the stylet was removed, and the needle was advanced through the placental disk. This created a “core” biopsy collected in the empty bore of the needle. A 5 mL syringe containing 1 mL of warmed, heparinized saline (5 units/mL) was then attached to the end of the needle, and the needle was moved back and forth within the placental disk 4 to 6 times while applying 3–4 mLs of negative pressure suction via the attached syringe. Altering the angle of the needle slightly with each advancement maximized the collection of aspirated villous tissue.

Biopsy samples were transported to the laboratory to obtain a wet weight prior to processing as follows: (1) flash frozen in liquid N_2_ for storage at −80 °C, (2) fixed in 10% neutral-buffered formalin for 24 h prior to transfer to 70% ethanol for paraffin embedding, or (3) washed in buffer for organoid isolation (described below).

### 2.4. Histology

Histological sections were cut from paraffin-embedded blocks at a thickness of 5 µm. Slides were heated to 60 °C for 1 h prior to being dewaxed in xylenes followed by ethanol gradient washes to rehydrate the tissue. After rehydration, the slides were stained using hematoxylin (Epredia, Kalamazoo, MI, USA, 72804) for 1 min, bluing buffer (Fisher Healthcare, Waltham, MA, USA, 220-106) for 1 min, and eosin (Fisher Healthcare, 220-104) for 30 s. The slides were immediately cover slipped and allowed to dry before digital imaging and review.

### 2.5. RNA Isolation

CVS biopsy samples were stored at −80 °C until RNA isolations were performed via phenol/chloroform separation using TRIzol reagent (Invitrogen, Carlsbad, CA, USA, 15596026). Immediately after thawing, 250 µL of lysis buffer (50 mM Tris pH 8.0, 100 mM EDTA, 0.5% SDS) was added and tissue was homogenized [24] before transferring to a clean tube. A series of 10 min incubations followed additions of 500 µL TRIzol, 250 µL TRIzol, and 200 µL of chloroform at room temperature with sample inversion. Samples were then spun at 4 °C at 21,300× *g* for 5 min, with the aqueous then layer drawn off and transferred into 500 µL of isopropanol with 1 µL of linear acrylamide carrier (AM9520) and incubated. Samples were spun at 4 °C at 21,300× *g* for 10 min and subsequently washed twice in 350 µL 80% ethanol, with spinning at 21,300× *g* at 4 °C for 5 min. Excess ethanol was removed, and the pellets were dried before eluting in 40 µL of nuclease-free water (ThermoFisher Scientific, Waltham, MA, USA, J71786.AP).

RNA concentrations and purity were measured via an Invitrogen Nanodrop instrument. If 260/280 ratios were not satisfactory (<1.8), the samples were cleaned as follows: nuclease-free water was added as needed to bring volumes to 100 µL. Then, 50 µL of 7.5 M NH4Ac and 200 µL of isopropanol were added to each sample to precipitate RNA and were incubated for 10 min at room temperature. Samples were spun at 21,300× *g* for 10 min, and supernatant was drawn off, followed by one wash with 80% ethanol. The supernatant was removed, and the pellet was air-dried for 10 min at room temperature. Pellets were dissolved in 40 µL of nuclease-free water and a Nanodrop Spectrophotometer was used to confirm purity.

### 2.6. Organoid Isolation

The isolation method described in brief here was scaled down from the full published protocol for rhesus macaque trophoblast organoids generated from whole placenta following first trimester delivery [25]. Organoid isolation was performed using serial CVS tissue samples from one animal at three gestational ages (G49, G72, and G106). Immediately following CVS, tissue was placed in pre-warmed wash buffer for <1 h. Homogenate was digested in 0.25% Trypsin-EDTA for 5 min at 37 °C with shaking before being strained and quenched with digest stop buffer. Undigested material in the strainer was transferred into collagenase V solution (1 mg/mL) and digested for an additional 5 min for maximum yield. All digest homogenates were quenched with digest stop buffer before being centrifuged at 600× *g* for 6 min to pellet cells. Cell pellet was washed once in DMEM/F12. Cells were then centrifuged and debris was removed, with 3 washes in DMEM/F12. Cells were suspended in Cultrex reduced growth factor basement membrane extract, and type R1 (R&D Systems, Minneapolis, MN, USA, 3433-005-R1) and 25 µL droplets were seeded into a 48-well plate. The Cultrex domes were given 15 min to polymerize at 37 °C before being overlaid with 250 µL of fresh organoid media.

## 3. Results

### 3.1. Pregnancy Assessment with CVS

**Figure 2:**
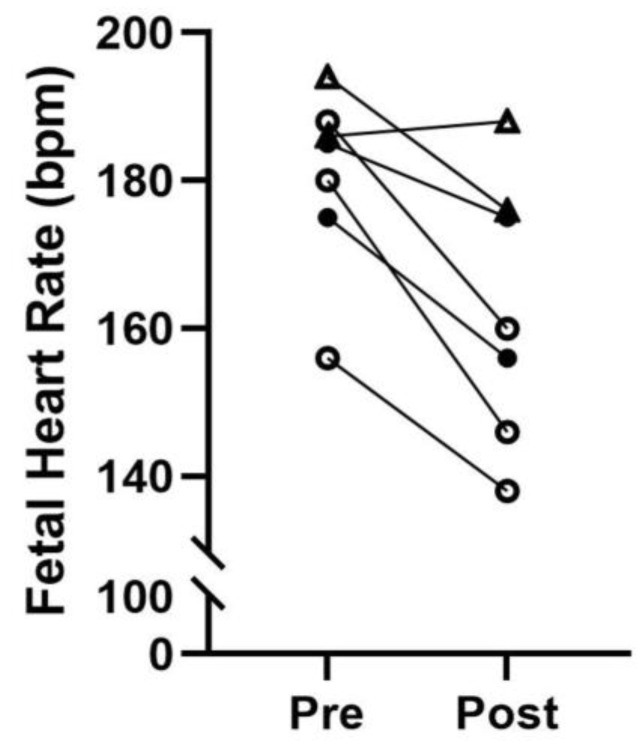
Fetal monitoring. Fetal heart rate prior to CVS (Pre) and post-procedure (Post). T1, G40–49: open triangles; T2, G63–72: closed circles; T3, G97–106: open circles; bpm: beats per minute.

The use of a simulation model prior to hands-on work in animals provides an opportunity to develop the necessary skills for performing the procedure and reduces the risk of adverse outcomes. Following simulation training, CVS was performed at three timepoints across gestation (ranging from G40 to G106) in each of the three rhesus macaques. At the time of CVS, fetal heart rate was measured by ultrasound and data were available for seven out of nine pilot studies (Figure 2).

### 3.2. CVS Biopsy Yields and Tissue Integrity

Tissue yields are reported in Table 2 and varied based on gestational age, operator experience, and placenta position. Depending on biopsy size, the samples were used for several purposes intended to provide quality control measures and assess suitability for in vitro analysis.

**Figure 3:**
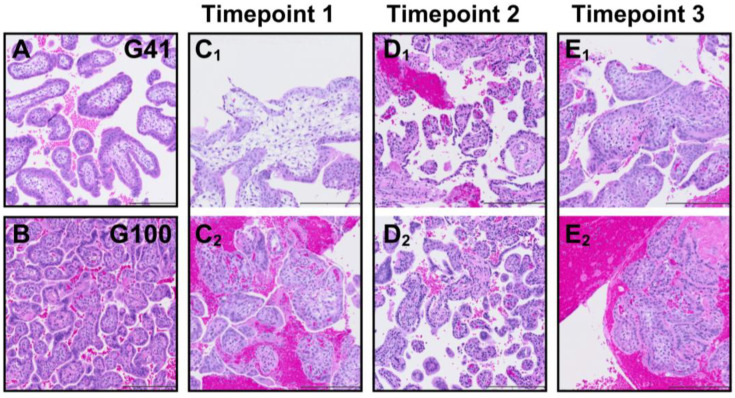
Placental histology. Representative H&E-stained placenta sections from whole placenta samples collected following surgical delivery at G41 (**A**) and G100 (**B**) for comparison with CVS biopsy samples from two animals per timepoint (Timepoint 1: G40–49 **C_1_**,**C_2_**; Timepoint 2: G63–72 **D_1_**,**D_2_**; Timepoint 3: G97–106 **E_1_**,**E_2_**). Scale bar is 200 µm.

The technique of obtaining biopsies via needle aspiration increases the risk of shearing the tissue under suction pressure and the needle bore imposes tissue sampling size constraints. Representative tissue sections from whole placentas collected following c-section delivery at G41 and G100, respectively (Figure 3A,B), provide comparison samples for histological assessment. Appropriately, we demonstrate more immature villi in the early placenta (Figure 3A,C) and no evidence of loss of tissue integrity in any of the CVS-obtained samples (Figure 3C–E). Note the increased presence of red blood cells (RBCs) and evidence of clots in some of the CVS-obtained samples. Technical note: An RBC lysis treatment can be added as an additional incubation between the transfer of biopsies from heparinized saline and fixative for histology or wash buffer for organoid isolation. For RBC lysis, tissue is incubated in buffer prepared in sterile water (10X RBC Lysis Buffer #00-4300-54, Invitrogen eBioscience) for 5 min with physical disruption every minute in light-protected conditions.

### 3.3. CVS Biopsy RNA Quality

RNA quality and yields for one biopsy sample per each of the three animals are reported in Table 3. Due to a technical issue not specific to the study, a clean-up step was required for each sample to improve purity and obtain a 260:280 ratio in the acceptable quality range. This led to a loss of RNA yield shown in the pre- versus post-RNA clean-up concentration values (Table 3). In the sample from animal C, the final yield was small but still usable for cDNA conversion.

### 3.4. CVS Biopsy-Derived Placenta Organoids

**Figure 4:**
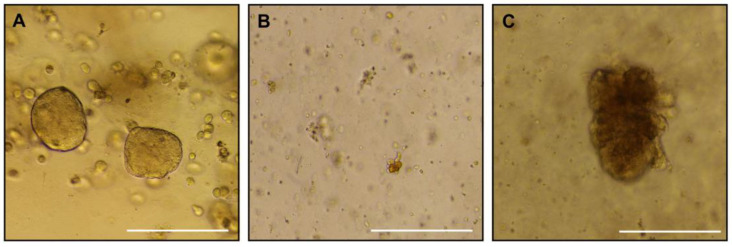
Placental organoids. Brightfield images of placenta trophoblast organoids isolated from CVS samples collected at G49 (**A**), G72 (**B**), G106 (**C**). Scale bar is 270 µm.

Placenta samples from animal C were used for trophoblast organoid isolation at each CVS timepoint, G49, G72, and G106 (Figure 4A–C). We were successful in isolating organoids at G49 that could be passaged to expand them in culture conditions. At the two later timepoints, organoid isolation was achieved but they remained sparse and failed to propagate well despite an extended time in culture.

## 4. Discussion

The use of ultrasound-guided needle procedures to collect villus biopsies has tremendous potential to facilitate longitudinal studies in continuing pregnancies. It could reduce the overall number of animals needed for pregnancy studies and expand the information obtained from each animal. This provides new opportunities for mechanistic studies in our NHP models of placental perturbation such as chronic maternal high fat diet [11,26] or Zika virus infection [6]. Moreover, the use of CVS techniques enables correlation of placental function in pregnancies that end in natural delivery (where placental collection is not feasible) with longer-term offspring health outcomes. In some instances, this sampling capability may negate the need for surgical deliveries where maternal infant repairing and extensive primate wellbeing behavioral specialist efforts are needed for mother rearing success. One notable limitation is the feasibility of performing CVS biopsies during early gestation (prior to G40), as the rhesus macaque placenta can be less than 0.2 cm in thickness, significantly increasing the technical challenge and introducing unnecessary risk of fetal developmental defects and miscarriage.

In this pilot, the veterinary surgeons only required two simulation training opportunities prior to performing CVS in pregnant females but for others, developing competency will be at the discretion of the expert observer and will in part be based on the prior experience of the trainee with ultrasound-guided needle aspiration procedures. Although this was not pursued in our pilot, a strength of this training model is the flexibility to trial more complex scenarios in the future. This might include the addition of solid objects to the pastry bag to simulate ribs and pelvis, positioning the tofu in a more difficult to reach area, or using a thinner piece of tofu to represent an earlier gestation placenta.

The tissue yields were small in our early CVS attempts, which does limit the downstream sample use. The most likely explanation is operator proficiency, although there are technical nuisances that can affect the success. For example, the most ideal positioning and ultrasonographic view of the placenta does not always align with the aspiration technique that is most achievable due to ergonomics or normal rhesus anatomy. In addition, we were constrained by protocol approval that did not allow for more than two CVS attempts for animal wellbeing. However, yields generally improved with experience gained and in an additional three opportunities for continued practice in performing CVS in pregnant animals on a subsequent project, biopsy yields ranged from 150 to 233 mg at gestational ages ranging from 53 to 150 days. Most importantly, we have observed no spontaneous preterm delivery in any animals subjected to serial CVS procedures (maximum of three with a minimum recovery period of two weeks between events). In addition, fetal heart rate was monitored at the start and completion of each CVS procedure. We demonstrate a modest decrease in fetal heart rate with maternal sedation by inhaled isoflurane for the 10–20 min procedure, but values remained within normal range for all animals [27]. One subjective ultrasound observation made at the third CVS timepoint was the presence of multifocal hyperechoic foci within the placenta, which is a typical appearance of mature, near-term placentas due to calcifications that form within the tissue. Early calcification can also be indicative of vascular disruption [28]. Understandably, some evidence of injury would be anticipated after the introduction and passing of a needle through the tissue. Importantly, the placenta has adaptive capabilities which allow it to tolerate a certain degree of insult and still have the ability to repair itself and maintain the pregnancy [29]. This natural occurrence of placental plasticity lends itself to the use of CVS and the expansion of longitudinal studies with minimal detrimental impact to pregnancy outcomes in the NHP.

The histological samples were an important indicator of biopsy use as one concern for in vitro placental structural analysis would be the possible shredding of tissue and loss of appropriate villous architecture. Our histology review demonstrates normal morphology of the tissue with one notable difference being increased visible blood contamination surrounding the villous tissue in some samples. Two protocol modifications that we introduced during the pilot were to add heparin into the saline in the collection syringe and to perform an RBC lysis step if warranted following gross visual inspection of the tissue at the time of weighing. A subjective observation indicated that the use of lysis buffer improved the quality of the tissue by reducing RBC contamination. Although this was not quantified, the lysis buffer did not appear to damage the tissue.

A technical issue resulted in the need for clean-up in all RNA samples. This was unrelated to the type of starting material (i.e., biopsy vs. post-delivery tissue) and would not be anticipated in future sample processing. Despite this minor setback, from the CVS biopsies, we were able to generate good quality RNA in quantities sufficient to allow gene expression studies. Another alternative, not trialed here, would be to isolate protein, which seems feasible with any sample that exceeds ~50 mg as the amount of starting material for a protein extraction protocol.

One rationale for the use of CVS, specific to the research needs of our team, was to test the ability to isolate placental trophoblast organoids from the biopsy samples. The first trimester is the period of pregnancy in which the highest proportion of cytotrophoblast cells, the stem cell of the placenta, are bipotent and have the potential to differentiate into different phenotypes [30]. Until now, development of NHP trophoblast organoids has required first trimester termination of pregnancy and a cesarean section surgery to allow the collection of the whole placenta [25]. In this pilot study, we were able to achieve organoid isolation from CVS-obtained biopsies. However, we observed slower growth and overall poor propagation in the later timepoints. It is possible this was due to a technical error, specifically the initial plating density may have been too sparse, which hindered the growth in our regular culture conditions; this can be investigated in future CVS culture attempts with further protocol refinement. Nonetheless, despite the less favorable outcome in the later CVS samples, the G49 biopsy resulted in successful organoid generation, which is important as the first trimester is the timeframe of greatest interest for our in vitro investigations. A second potential difficulty is that CVS-derived organoid cultures are susceptible to maternal gland contamination due to the introduction of the needle through the maternal uterine layers, which results in a less pure trophoblast cell population in the biopsy sample. This presents a challenge for the further development of CVS-generated trophoblast organoids. Maintenance of the stylet within the spinal needle during penetration of maternal tissues with removal after the placenta has been penetrated reduces contamination of the placental sample. However, additional troubleshooting within the sampling technique and the possible addition of steps within the isolation protocol to purify the preparations are needed to minimize contamination.

## 5. Conclusions

In conclusion, here we have provided training guidance to practice performing CVS procedures with demonstrated success in obtaining good quality placenta biopsies from pregnant NHPs. We suggest that the use of minimally invasive CVS in the research setting has the potential to enhance longitudinal studies and reduce the number of animals needed to conduct pregnancy investigations.

## Figures and Tables

**Table 1:** Maternal age and weights at time of CVS procedures.

Animal	Age (Years)	Timepoint 1	Maternal Weight (kg) Timepoint 2	Timepoint 3
A	5	6.7	6.85	7.75
B	6	8.15	8.35	8.95
C	6	5.35	5.45	6.00

**Table 2:** Tissue wet weights for CVS-obtained biopsies at timepoints 1 (G40–49), 2 (G63–72), and 3 (G97–106) for three animals (A–C).

Animal	Timepoint 1	Timepoint 2	Timepoint 3
A	66 mg	40 mg	15 mg
B	35 mg	13 mg	120 mg
C	n/a ^1^	118 mg	116 mg

^1^ Wet weight not recorded.

**Table 3:** RNA concentrations pre- and post-RNA clean-up with the final 260:280 ratio values for the three animals (A–C).

Animal	Pre	Post	260/280
A	893 ng/µL	201 ng/µL	1.92
B	1089 ng/µL	628 ng/µL	2.06
C	437 ng/µL	17 ng/µL	2.01

## Data Availability

The original contributions presented in this study are included in the article. Further inquiries can be directed to the corresponding author.

## Abbreviations

The following abbreviations are used in this manuscript:

CVS	Chorionic Villus Sampling
NHP	Non-human Primate
NAMs	New Approach Methods
IACUC	Institutional Animal Care and Use Committee
G	Gestational Day
